# Arboviral Bottlenecks and Challenges to Maintaining Diversity and Fitness during Mosquito Transmission

**DOI:** 10.3390/v6103991

**Published:** 2014-10-23

**Authors:** Naomi L. Forrester, Lark L. Coffey, Scott C. Weaver

**Affiliations:** 1 Institute for Human Infections and Immunity, Department of Pathology, University of Texas Medical Branch, Galveston, TX 77555-0610, USA; E-Mail: sweaver@utmb.edu; 2 Center for Vectorborne Diseases and Department of Pathology, Microbiology and Immunology, School of Veterinary Medicine, University of California, Davis, CA 95616, USA; E-Mail: lcoffey@ucdavis.edu

**Keywords:** bottlenecks, evolution, arboviruses, viral fitness, West Nile virus, Chikungunya virus, Venezuelan equine encephalitis virus

## Abstract

The term arbovirus denotes viruses that are transmitted by arthropods, such as ticks, mosquitoes, and other biting arthropods. The infection of these vectors produces a certain set of evolutionary pressures on the virus; involving migration from the midgut, where the blood meal containing the virus is processed, to the salivary glands, in order to transmit the virus to the next host. During this process the virus is subject to numerous bottlenecks, stochastic events that significantly reduce the number of viral particles that are able to infect the next stage. This article reviews the latest research on the bottlenecks that occur in arboviruses and the way in which these affect the evolution and fitness of these viruses. In particular we focus on the latest research on three important arboviruses, West Nile virus, Venezuelan equine encephalitis virus and Chikungunya viruses and compare the differing effects of the mosquito bottlenecks on these viruses as well as other evolutionary pressures that affect their evolution and transmission.

## 1. Introduction

### 1.1. Arboviruses

Arboviruses are arthropod-borne viruses that are transmitted between vertebrate amplifying hosts by mosquitoes, ticks and other biting arthropods that serve as vectors [1,2]. Their transmission cycles generally include an intrinsic incubation phase, when infection, replication and viremia occur in the vertebrate host, and an extrinsic incubation, when infection, replication, dissemination and ultimately shedding of virus into the saliva of the vector occur. Most arboviruses initiate infection of the vector after ingestion of viremic blood, which passes to the digestive tract (e.g., midgut for mosquitoes) and infects epithelial cells. An arbovirus must then replicate and spread into the hemocoel or open body cavity, where it gains access to a variety of secondary target organs including the salivary glands, where replication and deposition into the apical cavities of acinar cells can lead to inoculation into a host upon refeeding. Some viruses can also be transmitted mechanically (as opposed to biologically), via contamination of vector mouthparts during feeding, followed by infection of a new vertebrate host upon subsequent feeding without infection or replication in the arthropod, a process that is typically compared to a “flying pin.” However, mechanically transmitted viruses are not considered arboviruses *sensu stricto*. Some arboviruses can also be transmitted vertically via infected eggs, or venereally, or via nonreplicative viremia to co-feeding vectors. However, for the most part, these modes of transmission are thought to be insufficiently efficient to perpetuate circulation in the absence of some degree of horizontal transmission.

Arboviruses do not comprise a taxon because arthropod-borne transmission has evolved convergently several times, as indicated by the presence of arboviruses in several different RNA viral taxa, all of which also include non-arthropod-borne members: the alphaviruses (genus *Alphavirus*, one of two genera in the family *Togaviridae*); the flaviviruses (genus *Flavivirus*, one of three genera in the family *Flaviviridae*); the bunyaviruses, nairoviruses and phleboviruses (three of five genera in the family *Bunyaviridae*); the orbiviruses (one of nine genera in the family *Reoviridae*); the vesiculoviruses (one of six genera in the family *Rhabdoviridae*) and the thogotoviruses (one of four genera in the family *Orthomyxoviridae*) [3]. Only one DNA arbovirus is known, *African swine fever virus* (*Asfarviridae*: *Asfarvirus*), suggesting that the genetic plasticity and mutant swarm characteristics of RNA viruses are critical to maintaining the alternating vertebrate-invertebrate host cycle in nature (DNA viruses generally exhibit lower mutation frequencies than RNA viruses). Traditionally, it has been hypothesized that arboviruses are constrained genetically due to the “trade-off” in fitness that they must make when adapting simultaneously for infection and replication in both kinds of hosts. This view has been partially supported by experimental and retrospective studies of host range changes [4,5]. However, recent studies also suggest important effects of viral population bottlenecks that occur during the extrinsic incubation period.

In addition to their taxonomic diversity, arboviruses are ecologically highly diverse, utilizing vectors in four different orders of insects (Diptera, Anoplura, Siphonaptera and Hemiptera) and two families of ticks (Ixodidae and Argasidae) as vectors [2]. Vertebrate hosts of arboviruses are also highly diverse, with birds and mammals serving as the enzootic amplification and/or reservoir hosts for the majority of human-pathogenic arboviruses.

### 1.2. Bottlenecks

**Figure 1:**
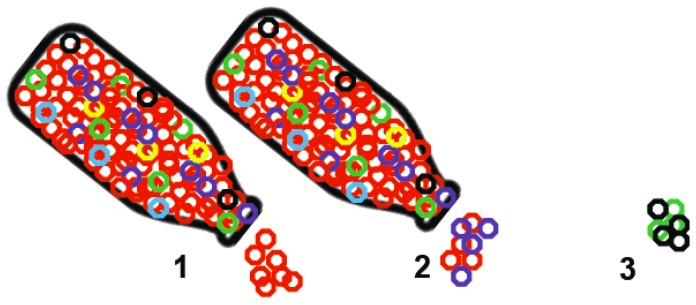
Effects of a bottleneck on virus populations, where virus variants are shown as colored circles: (1) Only the largest subpopulation is maintained after the bottleneck and viral variation decreases; (2) Virus variability decreases but a small amount of viral diversity is retained, and (3) Virus population diversity changes significantly due to random selection of small subpopulations and the dominant sequence is not perpetuated.

In evolutionary biology, a bottleneck is a stochastic or random event that decreases the number of viable replicating organisms in a population to a much smaller number, therefore reducing the effective population size. Following a bottleneck, one of two options will occur: the population will either go to extinction or recover to a larger effective population size. The genetic diversity of the population will be reduced regardless of the outcome [6]. This is illustrated in Figure 1, where the potential changes in diversity particularly associated with viruses can be seen.

Bottlenecks occur in all types of organisms and their populations including humans [7], the European rabbit [8] and various viral populations [6,9,10]. For viral populations the presence of bottlenecks should in theory lead to regular extinction events due to the lack of regular or efficient recombination of their genomes, which can restore the original master sequence following a bottleneck. A bottleneck in a viral population occurring during a life cycle would potentially increase the number of sub-optimally fit viral particles (because most mutations affect well adapted protein sequences and reduce fitness) being transmitted, just by chance. This conforms to the theory of Muller’s Ratchet, which predicts that asexual populations of organisms that periodically undergo population bottlenecks should tend to accumulate deleterious mutations unless sex or recombination intervene to allow efficient restoration of the wild-type (wt) or master sequence [11,12]. For viruses, bottlenecks can be experimentally induced by plaque-to-plaque transfer (initiating viral infection from a single virus particle forms one plaque), which simulates repeated natural bottleneck events and should be accompanied by fitness decreases as stochastic mutations are accumulated and the virus sequence becomes further and further removed from the consensus (average) sequence of the original virus. This has been experimentally demonstrated in many viruses [13,14,15,16,17,18,19,20]. Studies with foot-and-mouth disease virus (FMDV) by Domingo *et al.* [21] have shown that repeated bottleneck events induce a fluctuating fitness for approximately 20–40 passages, with fitness showing a fixed variation around the mean. Following the 20–40 passages the mean fitness decreases substantially but the oscillations around the mean increase give rise to greater diversity in fitness. Further, plaque-to-plaque propagation does not further decrease the mean fitness. After the virus has been subjected to 200 bottleneck events there is no increase in fitness, only a continued oscillation around the decreased mean fitness seen after 20–40 passages. Interestingly, the virus is never able to recover by compensating for this reduced fitness and this indicates a long-term, irreversible effect of bottlenecks on virus populations.

For arboviruses, the presence and effect of bottlenecks is especially important as the mosquito vector subjects the virus to several anatomical bottlenecks (reviewed in [4]). As arboviruses must infect all major compartments of the mosquito from infection of the midgut to dissemination into the hemocoel, to invasion of the salivary glands in order to be transmitted, they are constantly subjected to bottlenecks because most of these compartments are separated by basal lamina with limited viral penetration. The first evidence of this came when mosquitoes were infected with virus-like particles containing replicons expressing reporters such as green fluorescent protein. This allows the identification of the number of cells initially infected in the mosquito midgut. For Sindbis virus (SINV), West Nile virus (WNV) and epidemic VEEV and their associated mosquito vectors, a limited number of midgut cells were identified as initially infected using this method [22,23,24,25] indicating the presence of a significant bottleneck upon midgut infection. Interestingly, a similar experiment using the enzootic mosquito *Culex taeniopus* and an enzootic VEEV strain associated with this mosquito showed that almost the whole midgut was infected with the virus [26]. These findings show that the relationship between the mosquito and the virus influences the severity of the bottleneck upon entry into the midgut. Subsequent experiments with the same VEEV enzootic strains and the enzootic mosquito *C. taeniopus*, showed that the number of infected midgut cells was determined by the amount of virus ingested by the mosquito [9]. Further discussion of the bottlenecks associated with the best-studied viruses can be found below.

For tick-borne viruses, the only study of diversity was performed using Powassan virus (POWV), and this showed that POWV populations isolated from ticks contain little viral genetic variation. This suggests that, upon infection of *Ixodes scapularis*, POWV is subject to a high degree of purifying selection and bottlenecks that probably occur when the ticks moult to the next stage [27]. Further investigations of tick-borne viral infections are required to confirm the severity and timing of bottlenecks in other tick-borne viruses and the long-term effect on viral evolution.

Given the evidence presented above, it is likely that all arboviral infections are affected to some degree by bottlenecks. However, the severity and effect of these bottlenecks on the viral diversity is only just beginning to be uncovered and further experiments are required to determine the evolutionary pressures that such bottlenecks impose on arboviruses.

## 2. Transmission Cycles and Viral Diversity

Arboviral transmission cycles are complex and the viruses encounter different environments during their transmission cycles. Infection of the vertebrate host requires the virus to evade both innate and adaptive immune responses as well as to replicate to high enough titers to facilitate transmission to the next arthropod vector. It has long been postulated that the diversity present within the viral populations facilitates evasion of the immune system long enough for transmission. Certainly, viruses that have reduced diversity are no longer able to replicate as well within the host [28,29,30]. Experimental infections with Chikungunya virus (CHIKV) that had been mutated to reduce the number of mutant progeny via increased genome replication fidelity produced infections that were less virulent with reduced mortality associated with the infection [30]. These experiments confirm that the presence of intra-host viral genetic diversity (a diverse mutant swarm) is critical to the successful infection and spread within the multiple hosts required for horizontal arbovirus transmission in nature. Interestingly, increasing the level of mutant viruses, *i.e.*, making the virus a hyper-mutator, also decreases the infectivity of CHIKV [31], reducing the virulence and mortality. These results confirm that most viruses evolve an optimal level of replication fidelity and diversity, and any changes can result in compromised fitness.

Unlike vertebrates where the immune response is well understood, we are only just beginning to understand the immune responses associated with arbovirus vectors such as the mosquito and tick. Most of the work on insect immune systems has been done with *Drosophila* and is reviewed elsewhere [32]. For mosquitoes, the presence of an RNAi response has been well documented and there are three potential pathways; the small interfering RNA response (siRNA), the micro RNA pathway (miRNA) and the piwi-like RNA response (piRNA) [33]. Most of the work in arboviruses has been done with the siRNA response and is thus the focus of this review. The first indication of the presence of the siRNA response in mosquitoes was homology with *Drosophila* genes, and homologues of the important genes Dicer-2 (Dcr-2), R2D2 and Argonaute-2 (Ago-2) have been found in *Anopheles gambiae*, *C. pipiens* and *Aedes aegypti* [34,35].

In general, arboviruses infect mosquito cells without causing significant damage, with little evidence of the cytopathic effects (CPE), usually seen in vertebrate cells. This allows mosquitoes to remain infectious for their lifetime, compared to vertebrates that typically clear the infection from their systems or succumb to infection. Recent evidence suggests that this long-term vector infection is the result of suppression of the virus via siRNA responses. When the alphavirus O’nyong-nyong (ONNV) is used to experimentally infect *An. gambiae*, the virus reaches the salivary glands in 9 days. If the virus is co-infected with double-stranded ONNV RNA that stimulates the siRNA response, this leads to a slower infection with the virus increasing the time required to disseminate and reach the salivary glands. However, when ONNV is co-infected with siRNAs that inhibit the RNAi response, the virus spreads more rapidly and achieves higher mosquito titers, even leading to increased mosquito mortality when *An. gambiae* is infected with ONNV [35,36]. This shows that virus infection of the mosquito is not inherently benign as was traditionally thought, but is kept in check by the mosquito immune response, allowing the mosquito and virus to survive long enough for the virus to be transmitted. The importance of the siRNA response in suppressing viral replication has been shown by the incorporation of the Flock house virus (FHV) protein B2 into the alphavirus Sindbis virus (SINV). The B2 protein of FHV is a suppressor of the insect immune response [37]. Upon infection with SINV containing the B2 protein of *A. aegypti* cell cultures or *in vivo* mosquitoes results in increased infection, dissemination and high viral titers and increased mortality [38]. Thus this experiment confirms that alphaviruses are virulent to mosquitoes in the absence of a competent innate immune system.

Given that arboviruses are subject to antiviral pressures from the mosquito as well, the presence of viral diversity appears to be more important in the mosquito vector than was previously recognized. Recent work examining the effect of the RNAi response on the production of siRNAs from the virus has shown that there are areas of the viral genome that disproportionately produce the viral RNAs (viRNAs) that trigger the immune response. Studies with SINV show that these viRNAs are generated from both the positive and negative-strand of the genome, though mainly from the positive strand [39,40]. These areas of diversity were mapped to different regions in the positive strand compared to the negative strand. Studies with WNV showed that the hotspot genomic regions exhibited higher levels of diversity [41], suggesting that the presence of the immune response affects arboviral diversity.

Arboviral genetic diversity is therefore an essential strategy for the virus to ensure survival and represents a new and exciting avenue of virus evolution research. More work is needed to examine the interactions between the RNAi response and viral diversity. Additionally, the impact of the repeated bottlenecks described above needs to be investigated to give a complete picture of the complex interplay between the mosquito and the virus that facilitates viral transmission without adverse effects to the mosquito.

## 3. Evolutionary Pressures on Arboviruses and Fitness Potentials

Arboviruses are unique in that they obligately cycle between vertebrates and invertebrates, unlike most RNA viruses that use just one or several closely related host species. The need to replicate in two different host types imposes special selective constraints on arboviruses and shapes their evolution. Invertebrate and vertebrate hosts are believed to present conflicting challenges that limit adaptation to either host alone by imposing fitness costs where adaptations are antagonistic [42]. As evidence of these challenges, arboviruses isolated from nature show less genetic variation than predicted by their intrinsic mutation rates [43,44,45]. Many experimental evolution studies with CHIKV, EEEV, Rift Valley Fever virus, SINV, VEEV, Vesicular stomatitis virus, and WNV, (reviewed elsewhere [4,46]) where one host is artificially removed from alternating transmission via serial passage, illustrate the trade-offs inherent to dual-host cycling. These studies reveal three general patterns of arbovirus evolution: (i) fitness gains after serial passage and fitness losses in bypassed hosts (with some exceptions); (ii) reduced fitness in new hosts not previously involved in the transmission cycle, and (iii) fitness increases in passaged hosts after alternating (invertebrate-vertebrate) passage. Together these studies show that constraints on fitness differ in invertebrate *versus* vertebrate hosts and can be virus‑specific, but that arbovirus fitness is not severely limited by alternating between vertebrate and invertebrate hosts.

Although recent work has focused on defining the viral genetic determinants of emergence and host range changes (reviewed elsewhere [4,47]), knowledge of the composition and role of arbovirus heterogeneity in cycling and vector infection is scant. RNA viruses, including nearly all arboviruses, exist as heterogeneous populations of highly similar genomes, together called mutant swarms. Heterogeneity arises from error-prone replication due to the inability of the viral polymerase to correct mistakes, in concert with rapid and exponential population growth. However, arboviruses have historically been treated as clones of the same genomic RNA; this view is simplistic in that it ignores the mutant swarm and its possible phenotypic roles. Characterizing the mutant spectrum in large and heterogeneous populations requires extensive sequencing that was labor intensive until the recent development of next generation sequencing, which allows full genome coverage at high depth with relatively little work [48]. An increasing number of studies are employing this technology to characterize mutant spectra.

## 4. Case Studies

The three viruses studied below are the most well studied arboviruses in terms of their evolution and transmission. West Nile virus has been studied in detail since its first identification in the U.S. in 1999. Since then, there have been numerous studies on its evolution in both avian and mosquito hosts and it remains the best understood flavivirus in terms of how the virus and the hosts interact. CHIKV and VEEV both belong to the genus *Alphavirus*. Of these VEEV has been of historic interest since the 1930s due to its periodic reemergence as an epidemic virus and the ability of the virus to spread rapidly throughout Central and South America, and even to the U.S., during epidemic outbreaks [49]. Chikungunya recently emerged from an enzootic cycle in Africa to become nearly pandemic, causing a millions of cases of human disease in the past few years. Partly because of the historic interest in VEEV and the recent epidemics of CHIKV, the transmission cycles of these two viruses are well understood and, as a result, it is possible to study their evolution in detail, where other less-well understood systems would be more challenging.

### 4.1. West Nile Transmission and Bottlenecks

West Nile virus, has been well studied since its arrival in New York in 1999 [50], and the evolution of the virus has been the subject of many papers [51,52,53,54] showing numerous genetic changes. Both the vector and host influence the evolution of the virus and therefore understanding the diversity of the virus and the effect of the diversity on the host will increase the insight into this important pathogen.

Studies of WNV have shown that it, like all RNA viruses, exhibits diversity within infected hosts. Evidence for the presence of diversity has been identified in both the mosquito vector and the avian host. However, experimental quantification of the amount of variation suggests that there is more diversity in vectors compared to avian hosts [55]. Experimental evolution studies have shown that when the virus is artificially released from the need to infect both hosts, there is a lesser diversity in mosquitoes compared to chickens when passaged in chickens, whereas when both were passaged in mosquitoes the amount of diversity increased [56] (opposite to the pattern observed for VEEV [57]). Increased diversity in mosquitoes may stem from the need to avoid the RNAi response, described above.

For WNV two experiments were conducted, the first showed that upon infection of a mixture of five variants in roughly equal quantities (much like the VEEV work described below) the *C. pipiens* mosquitoes do not appear to exhibit bottlenecks, showing no reductions in the number of viral variants between the midgut, the hemocoel and the salivary glands. Instead, the amount of diversity decreases over time in all vector tissues and organs over a 21-day extrinsic incubation period. This decrease appears to be stochastic, with no one variant consistently being selected [58]. However, when a second experiment was performed with eight variants with differing proportions, there was a significant decrease in the variants isolated from the midgut compared to the original bloodmeal and a decrease in the number of variants in the salivary secretions compared to the midgut. In addition, a temporal decrease was observed in addition to the bottlenecks. This temporal decrease was not observed in the VEEV experiments (see below) and may be a result of the vector-virus relationship in this particular transmission cycle. It is also possible that the presence of RNAi reduces diversity from day seven to day 21 post-infection in *C. pipiens* mosquitoes and WNV. Further study of other systems is needed to assess the relative changes in temporal *versus* anatomical reduction in variants in different systems.

### 4.2. Venezuelan Equine Encephalitis Virus

**Figure 2:**
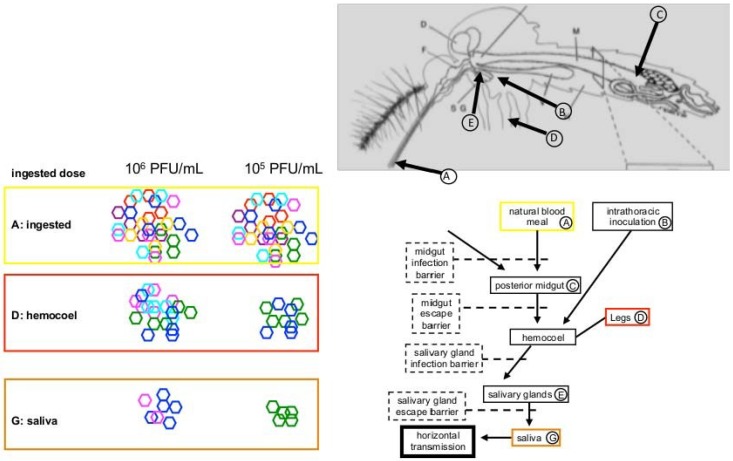
Changes in Venezuelan equine encephalitis virus populations after anatomical bottlenecks in *Culex taeniopus* mosquitoes fed mixtures of eight barcoded virus variants colored circles). Variant diversity decreased at dissemination and in saliva and the number of variants transmitted in saliva was lower in mosquitoes that ingested lower viral doses.

The presence of bottlenecks in VEEV during mosquito infection has been studied using subtype IE strains and the associated enzootic vector *C. taeniopus* [9] (illustrated in Figure 2).

A series of eight viruses marked in the nsP2 gene with synonymous mutations was created, all with equal mosquito fitness *in vitro* and *in vivo*. When all eight marked viruses were fed high bloodmeal titers (10^6^ pfu/mL) to cohorts of mosquitoes, they all present in the infected mosquito midguts. However, when the hemocoel was tested for the presence of the marked viruses there was a significant reduction in the number of marked clones, suggesting the presence of a bottleneck upon virus escape from the midgut into the hemocoel. Effective population size measures estimated that ≈50 infectious virus particles escaped from the midgut into the hemocoel. However, when mosquitoes were fed with lower VEEV titers (10^5^ pfu/mL) the number of marked viruses detected in the midgut decreased, indicating the presence of a bottleneck upon midgut entry. Midgut infection is therefore dependent on the initial concentration of ingested virus, and in natural transmission cycles probably represents a significant bottleneck as most rodents have viremia titers lower than 10^4^ pfu/mL during infection with VEEV IE [59]. At the lower (10^5^ pfu/mL) titer there was still a bottleneck associated with escape from the midgut, and the number of particles calculated to escape was approximately two. Following injection of the virus via the intrathoracic route to artificially bypass midgut infection, evidence of a third bottleneck was detected when VEEV infects the salivary glands from the hemocoel. Although transmission experiments were conducted, only three transmission events were documented, so it was not possible to determine a consistent bottleneck associated with vertebrate infection. Therefore it appears that for VEEV, the mosquito is the major source of bottlenecks, and these are associated with the anatomical barriers in mosquitoes, unlike WNV where the reduction in viral genetic diversity is associated with time.

### 4.3. Chikungunya Virus

Chikungunya virus is a re-emerging alphavirus that causes febrile disease with arthritis and has recently expanded from Africa to Indian Ocean Islands, Asia, Europe and the Americas [60]

The precedent for CHIKV studies came from an *in vivo* study with VEEV that detected significant fitness increases after serial single-host passages but no genome-wide consensus changes [57], suggesting a phenotypic role for minority genomes in the mutant spectrum. Subsequent studies with CHIKV therefore focused on characterizing the mutant spectrum after passage to understand how obligate host cycling shapes the viral population. CHIKV serially passaged in cells showed higher fitness in novel cell types and enhanced neutralization and antiviral compound resistance, changes that were associated with increased genetic diversity [61]. By comparison, alternating passages between cell types restricted CHIKV fitness and diversity increases, where mutations beneficial or neutral in both hosts were preserved to retain fitness in alternating cycling [61]. In parallel with earlier studies that did not involve sequencing, these results suggest an evolutionary trade-off between maintaining fitness for alternating host cycling and adaptability, where maximum adaptability is possible via augmented population diversity. Diversity probably helps virus populations by making them more likely to contain advantageous mutations when novel selective pressures are applied.

Recent studies with CHIKV [62] employing next generation sequencing technologies reveal that intra- and inter-host population infection and transmission dynamics in vectors are complex. Even though they can overcome anatomical bottlenecks at the midgut and salivary glands, probably by regenerating diversity via replication, the mutant spectra ingested by bloodfeeding vectors are not the same as the spectra they transmit. Moreover, certain transmitted minor variants with E1 glycoprotein mutations augment infectivity and transmissibility by vectors as well as pathogenesis in mice. This study also demonstrated that mutations with epidemiological relevance, including the E1 residue 226 alanine-to-valine substitution that promoted expansive epidemics in Indian Ocean Islands and Asia [63,64], can be detected in vector saliva during standard experimental transmission studies only using deep sequencing, highlighting the potential for detection of epidemic variants in laboratory studies before they explode in outbreaks.

## 5. Conclusions

Since the pioneering work of Muller [11] the potential effects of population bottlenecks on population fitness have gained more and more experimental support, especially using viruses as model systems due to their large population sizes, high mutation frequencies, and experimental tractability. Recently, arboviruses have played a central role in these studies because most undergo recombination infrequently (and recombination was hypothesized by Muller to compensate for mutational load following repeated population bottlenecks). *In vitro* studies have generally showed that arboviruses are subject to fitness declines following repeated bottlenecks (typically created with either limiting dilutions or plaque-to-plaque transfers). Population bottlenecks *in vivo* during vector infection have been shown using experimentally barcoded arboviral populations and deep sequencing of wt viral populations analysed in different compartments at several stages of vector infection and transmission. However, somewhat surprisingly, although these bottlenecks are apparent in several arbovirus-vector systems, there is little or no evidence that they affect fitness or even genetic stability in experimental settings or in nature. Key questions requiring further study include: (1) How do arboviruses recover their genetic diversity, which has been shown experimentally with high fidelity mutants to be critical for fitness and transmission [28], after population bottlenecks?; (2) Why do rates of nucleotide substitution for arboviruses in nature remain low, even for synonymous mutations that should not be constrained by purifying selection? (3) Do population bottlenecks constrain adaptive evolution and therefore modulate host range changes and arboviral disease emergence? Novel methods and tools including high throughput, deep sequencing and fidelity mutants generated recently for some arboviruses should facilitate future studies to address these fundamental questions.
