# Correction: Forrester, N.L.; Coffey, L.L.; Weaver, S.C. Arboviral Bottlenecks and Challenges to Maintaining Diversity and Fitness during Mosquito Transmission. *Viruses* 2014, *6*, 3991–4004

**DOI:** 10.3390/v6114422

**Published:** 2014-11-14

**Authors:** Naomi L. Forrester, Lark L. Coffey, Scott C. Weaver

**Affiliations:** 1 Institute for Human Infections and Immunity, Department of Pathology, University of Texas Medical Branch, Galveston, TX 77555, USA; E-Mail: sweaver@utmb.edu; 2 Center for Vectorborne Diseases and Department of Pathology, Microbiology and Immunology, School of Veterinary Medicine, University of California, Davis, CA 95616, USA; E-Mail: lcoffey@ucdavis.edu

In the original manuscript, Forrester, N.L.; Coffey, L.L.; Weaver, S.C. Arboviral Bottlenecks and Challenges to Maintaining Diversity and Fitness during Mosquito Transmission. *Viruses* **2014**, *6*, 3991–4004, Figure 1 contains an error, the third bottle was absent from the figure:

The correct figure should be:

**Figure 1:**
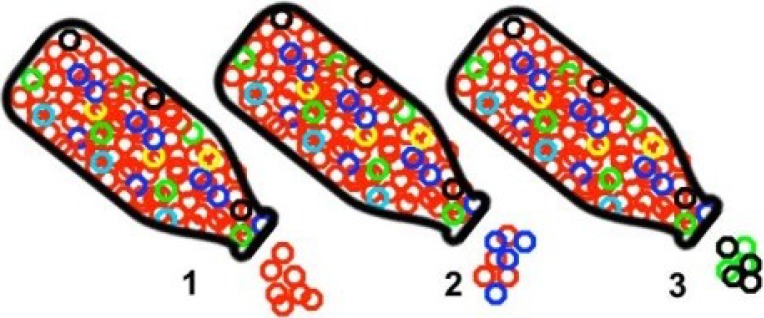
Effects of a bottleneck on virus populations, where virus variants are shown as colored circles: (**1**) Only the largest subpopulation is maintained after the bottleneck and viral variation decreases; (**2**) Virus variability decreases but a small amount of viral diversity is retained; and (**3**) Virus population diversity changes significantly due to random selection of small subpopulations and the dominant sequence is not perpetuated.
